# Application of Serious Games in Health Care: Scoping Review and Bibliometric Analysis

**DOI:** 10.3389/fpubh.2022.896974

**Published:** 2022-06-10

**Authors:** Yue Wang, Zhao Wang, Guoqing Liu, Zhangyi Wang, Qinglong Wang, Yishan Yan, Jing Wang, Yue Zhu, Weijie Gao, Xiangling Kan, Zhiguo Zhang, Lixia Jia, Xiaoli Pang

**Affiliations:** ^1^The School of Graduate, Tianjin University of Traditional Chinese Medicine, Tianjin, China; ^2^Nursing Department, Tianjin Medical University Chu Hisen-I Memorial Hospital, Tianjin, China; ^3^The School of Nursing, Tianjin University of Traditional Chinese Medicine, Tianjin, China; ^4^Dean's Office, Tianjin University of Traditional Chinese Medicine, Tianjin, China

**Keywords:** serious games, health care, rehabilitation, education, bibliometric analysis, scoping review

## Abstract

**Background:**

Serious games (SGs) as one kind of intervention that can improve the level of knowledge and change behavior to affect health outcomes has been increasingly applied in health care.

**Objective:**

Analyze hotspots and trends of the application of SGs in health care and provide reference and direction for further research in the future.

**Methods:**

The Web of Science (WoS) Core Collection database was used for extracting the literature on SGs in health care for the period from the database established to 11 October, 2021. Scoping review and bibliometric analysis were used to deeply analyze and visualize countries, categories of studies, annual study output, cited authors, cited journals, cited articles, and keywords of healthcare field.

**Results:**

A total of 1,322 articles were retrieved, then every articles' title and abstract were read one by one, and 795 articles were included after screening with an exponential increase in publication volume. The United States of America made the greatest contribution to global publications regarding SGs in health care. From the total, 20.8% of articles fall under the category of health care sciences services. The target groups were mainly concentrated in children (18.0%), youth (13.8%), the elderly (10.9%), adolescents (9.1%), and adults (3.4%). Baranowski T (*n* = 103 citations) is the most influential author, followed by Kato PM (*n* = 73 citations) and Desmet A (*n* = 58 citations). The top three cited journals were “*Plos One*” (*n* = 268 citations), “*Games for Health Journal*” (*n* = 209 citations), and “*Journal of Medical Internet Research*” (*n* = 197 citations), and the top three cited articles were “A meta-analysis of serious digital games for healthy lifestyle promotion,” “A Systematic Review of Serious Games in Training Health Care Professionals,” and “Video game training enhances cognitive control in older adults.” More and more studies focus on specific age groups, such as children, adolescents, and the elderly. The research hotspots and trends included “rehabilitation,” “medical education,” and “design.”

**Conclusions:**

The application of SGs in health care remains important areas for future research. “Rehabilitation,” “medical education,” and “design” reflected the latest research hotpots and future trends.

## Introduction

The term “serious games (SGs)” was first put forward by a famous American scholar in the book *Serious Games* in 1970 (1); and SGs are designed to achieve purposes other than entertainment (2). At present, there is no unified definition of SGs, and developers in different fields and different research have different understanding and definition of SGs. Special games are different from common games for entertainment that are added to at least one explicit reality simulation additional motivation, also called serious purpose (3). Special games have been applied successively to, but not limit to, military, management, education, medical and other fields. Especially in the field of health care, the application of SGs is increasing, like *Re-Mission* for adolescents and young adults with cancer (4), *Wii* for balance (5), *Elm City Stories* for prevention (6) and so on.

Special games have been applied in various clinical trials as an intervention and proved effective in a variety of diseases, such as cancer, asthma, mental health disorders and so on. For example, Bul et al. (7) implemented a multisite randomized controlled trial to determine the effects of SGs (called *Plan-It Commander*) on daily life skills of children with attention deficit and hyperactivity disorder (ADHD), with the result showing participants receiving SGs intervention compared to treatment-as-usual crossover group achieved significantly greater improvements on functional outcomes in daily life (*p* = 0.004). Meanwhile some systematic reviews demonstrated that SGs were effective in medical education (8, 9). The effects of SGs include many of the following aspects: Improving the level of knowledge (10), changing behavior (11), improving dexterity (12), reducing emotional problems (13), and so on. More and more researchers attach importance to SGs and apply them to clinical practice, and participants are also willing to accept them. The reason why SGs can attract people of all ages is that they have the following characteristics: Interaction, feedback, agency or control, identity, and immersion (14).

In the recent years, more and more scholars have summarized the application of SGs, including some quantitative studies, such as bibliometric analysis and systematic review, and qualitative analysis, such as scoping review. Schöbel et al. (15) presented a bibliometric analysis of existing literature on game concepts in digital learning and Hallinger et al. (16) conducted a bibliometric analysis on simulations and SGs in educating for sustainability, that both studies focused on the application of SGs in education. Also, some recent systematic reviews have reported the effectiveness of SGs in the fields of rehabilitation (17), psychotherapy (18), and education (19). There were several scoping review exploring SGs for rehabilitation in terms of stroke rehabilitation (20), upper extremity rehabilitation (21), musculoskeletal diseases rehabilitation (22), and so on. It is evident that this domain of SGs research is promising; consequently, more and more scholars direct their energies to this dynamic research field.

The purpose of our study is to summarize the application status of SGs in the field of health care, so as to put forward the research trend in the future. Bibliometric method has emerged as one of the most widely used methods to quantitively analyze some indicators that can reflect the research status and reveal trends in the scientific and applied fields, that are established as a scientific specialty (23). The interest of bibliometric analysis in understanding the current situation and trends for a certain field escalates recently (24). Consequently, we used bibliometric method to quantitively analyze general information, knowledge base, hotpots and trends about SGs in health care. And CiteSpace software is a good tool to complete bibliometric and visual analysis that is based on Java environment. In our study, the CiteSpace software is used to make visual and bibliometric analysis of cited authors, countries, cited journals, cited articles and keywords. However, more and more researchers focus on not only quantitative data, but also qualitative data. Therefore, our study combines scoping review and bibliometric analysis to comprehensively analyze the application status and research front of SGs in the field of health care.

## Methods

### Scoping Review

Scoping review, a form of knowledge synthesis, can examine the extent, range, and nature of the existing literature (25). Compared with systematic review, scoping review has two main differences: First, whether there is a well-designed theme, and scoping review tends to focus on broader topics. Second, whether to carry out the quality assessment of the literature, and scoping review is unlikely to evaluate the quality of the included literature (26). Our study was implemented under the methodological framework of Arksey and O'Malley (26), which included the following five stages: There were three stages in methods (identifying the research question, identifying relevant studies, and study selection) and two stages in results (charting the data; collating, summarizing, and reporting the results).

#### Identifying the Research Question

What is known from the existing literature about target groups, diseases, and future research directions of SGs in the field of health care?

#### Identifying Relevant Studies

An academic search of the Web of Science (WoS) core database was performed when it was established until 11 October, 2021, using the following keywords: TS = [(“serious gam^*^” OR “video game” OR “computer game”) AND (health^*^ OR care^*^)]. The WoS contains a large number of high-quality and influential magazines from all over the world and is an authoritative database covering all disciplines (27). The type of our search results was limited “article” and “review” and English articles only.

#### Study Selection

The following inclusion criteria were adopted: (1) Literature focused on SGs and (2) literature focused on healthcare field.

### Bibliometric Analysis

After extraction, the included literature was in accordance with the scoping review exported as plain text and transferred into CiteSpace for visual and bibliometric analysis. CiteSpace combines computational metrics and visual attributes of pivotal points, which can reduce cognitive burden. It allows researchers to easily identify pivotal points and emerging trends in a certain field (28). CiteSpace was used to create charts of cited authors, countries, cited journals, cited articles, and keywords. We set the CiteSpace parameters as follows: time span (1998–2022), years per slice (1), network nodes (cited author, country, cited journal, cited articles, keyword), and other options remain default. This study did not include people, and therefore there is no need for ethical approval.

## Results

### Scoping Review Results

**Figure 1:**
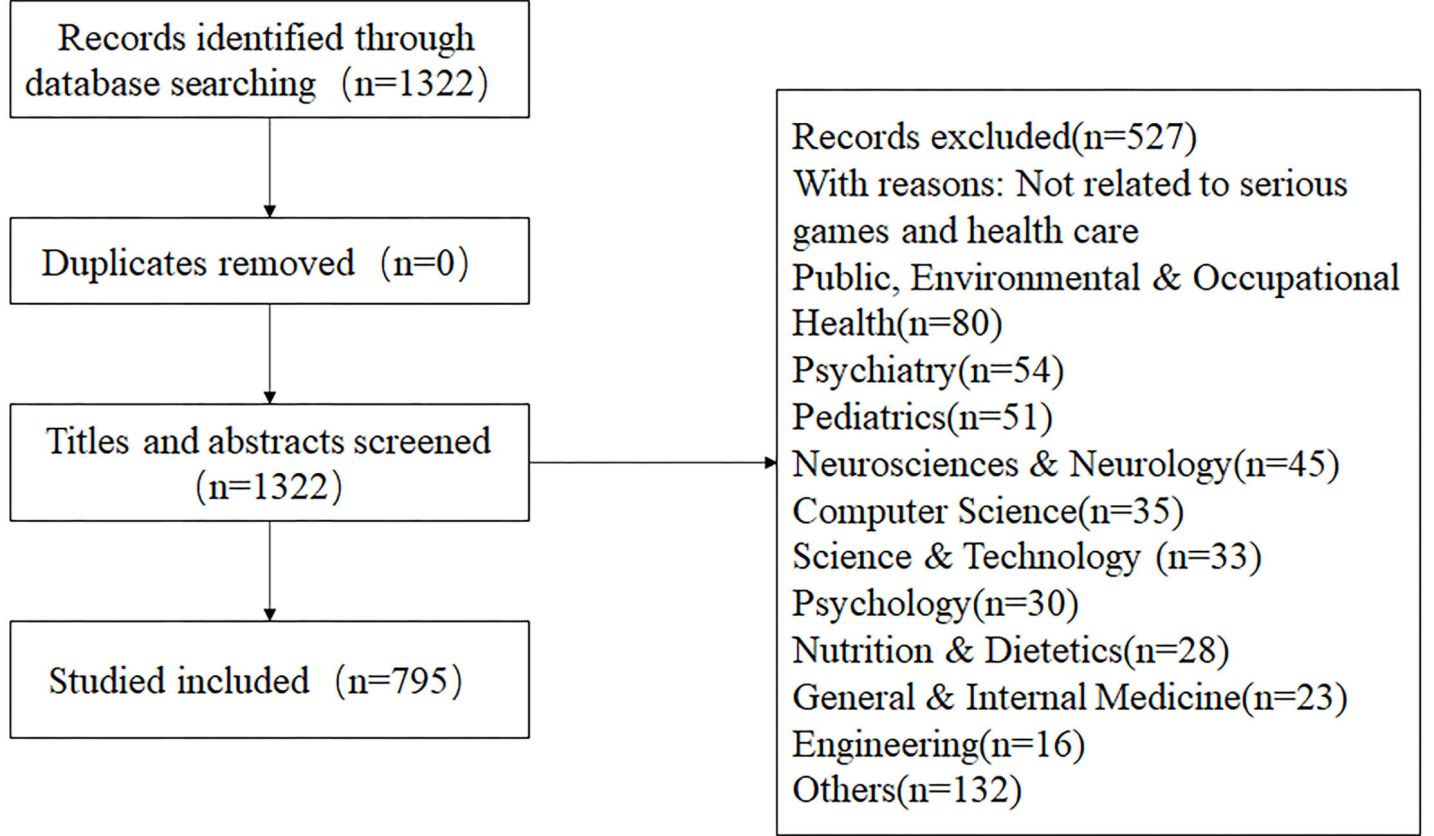
Flow diagram of study selection.

In our study, a total of 1,322 articles were retrieved, then every article's title and abstract were read one by one, and 795 articles were included after screening (see Figure 1).

#### Charting the Data

**Figure 2:**
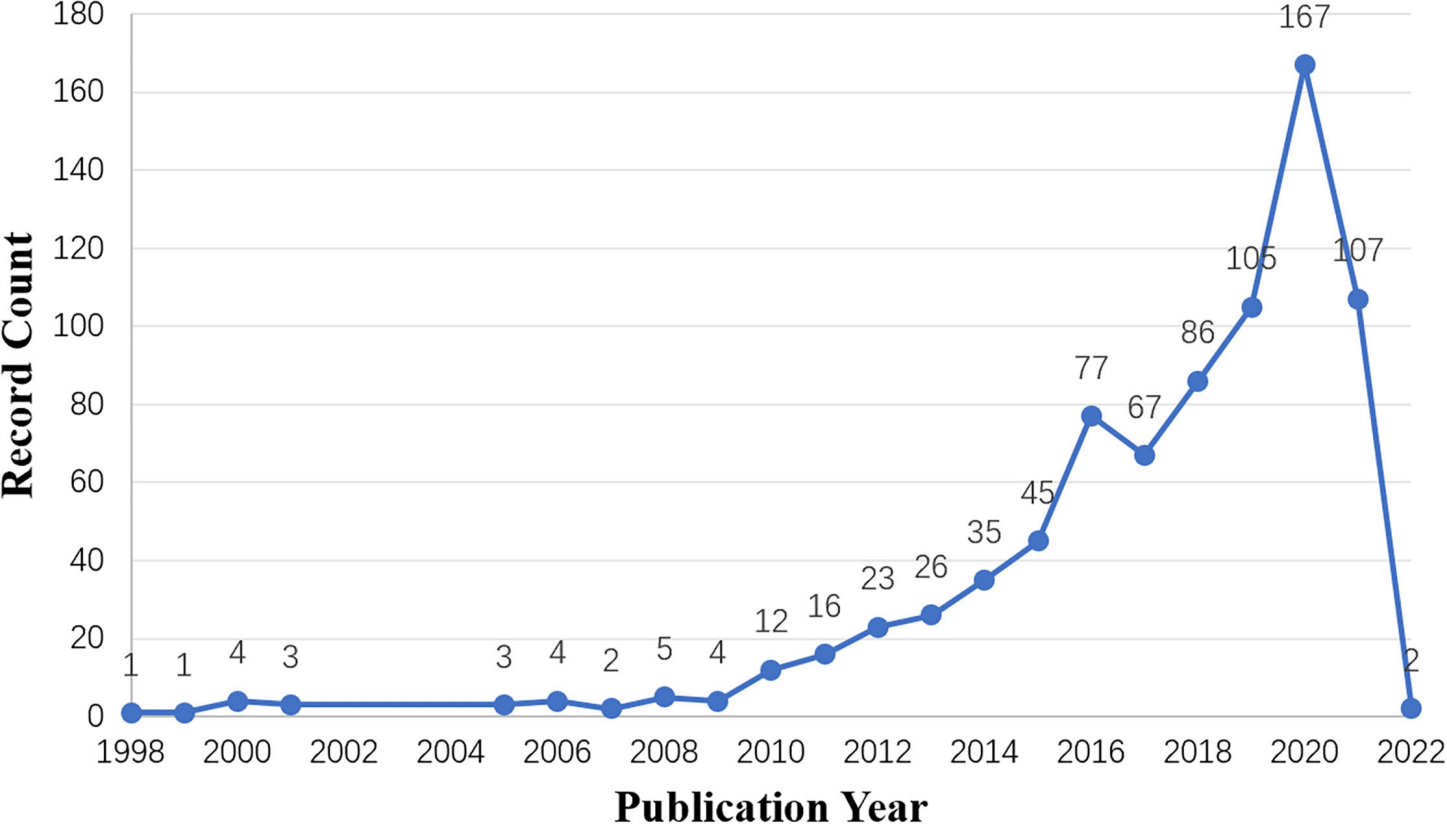
The annual articles output.

Characteristics of the eligible articles are presented in Table 1, including countries, articles' categories, target groups and disease, and annual article output are presented in Figure 2. As an important index, annual article output issued in a certain field reflects the research vitality in this field. Through the characteristics of included literature, we can clearly understand the application status and infer the trends of SGs in health care.

**Table 1:** Characteristics of eligible scoping review articles.

**Classification**	**Articles (*N* = 795)**	**%**
Countries of articles contributed^a^		
United States	218	27.4
Netherlands	82	10.3
England	80	10.1
Canada	79	9.9
Spain	70	8.8
Germany	49	6.2
Australia	47	5.9
Switzerland	41	5.2
France	37	4.7
Brazil	36	4.5
Categories of articles^a^		
Health care sciences services	165	20.8
Medical Informatics	164	20.6
Public environmental occupational health	118	14.8
Neurosciences	87	10.9
sRehabilitation	62	7.8
Psychiatry	58	7.3
Computer science information systems	45	5.7
Pediatrics	43	5.4
Geriatrics gerontology	42	5.3
Computer science interdisciplinary applications	41	5.2
Age groups^b^		
Children	143	18.0
Youth	110	13.8
The elderly	87	10.9
Adolescents	72	9.1
Adults	27	3.4
Disease ^b^		
Alzheimer's disease/Dementia/Cognitive Impairment	37	4.7
Mental illness	37	4.7
Stroke/Hemiplegia	31	3.9
Obesity/Overweight	21	2.6
Cancer/Tumor	18	2.3
Anxiety/Depression	16	2.0
Chronic disease	15	1.9
Diabetes	14	1.8
Pain	12	1.5
Multiple sclerosis	12	1.5

a *As the authors of an article may involve different countries and the types of an article may belong to multiple types, the column may not be equal to the total number of 795, and only the top 10 are shown in the table.*

b *We just listed the “age groups” and “disease” which were with more research literature.*

#### Collating, Summarizing and Reporting the Results

Table 1 reported the key characteristics of eligible articles, where we can see that the United States contributed 27.4% of the articles, ranking first with great advantage. 20.8% of articles fall under the category of health care sciences services, followed by medical informatics (20.6%) and public environmental occupational health (14.8%). The target groups were mainly concentrated in children (18.0%), youth (13.8%), the elderly (10.9%), adolescents (9.1%), and adults (3.4%). Special games are the most studied in Alzheimer's disease/dementia/cognitive impairment (4.7%) and mental illness (4.7%), followed by stroke/hemiplegia (3.9%).

As shown in Figure 2, the annual article output showed an obvious growth trend. From 1998 to 2020, the annual number of documents issued increased exponentially with an average of 36.1 articles per year, suggesting that more and more experts and scholars pay attention to the application of SGs in health care. The reason for the decrease in the annual article output from 2020 to 2021 may be that the retrieval time is 11 October, 2021, and the annual number of articles is incomplete. There are two articles in 2020 because some articles are published online in advance.

### Bibliometric Analysis Results

#### Cited Authors

**Figure 3:**
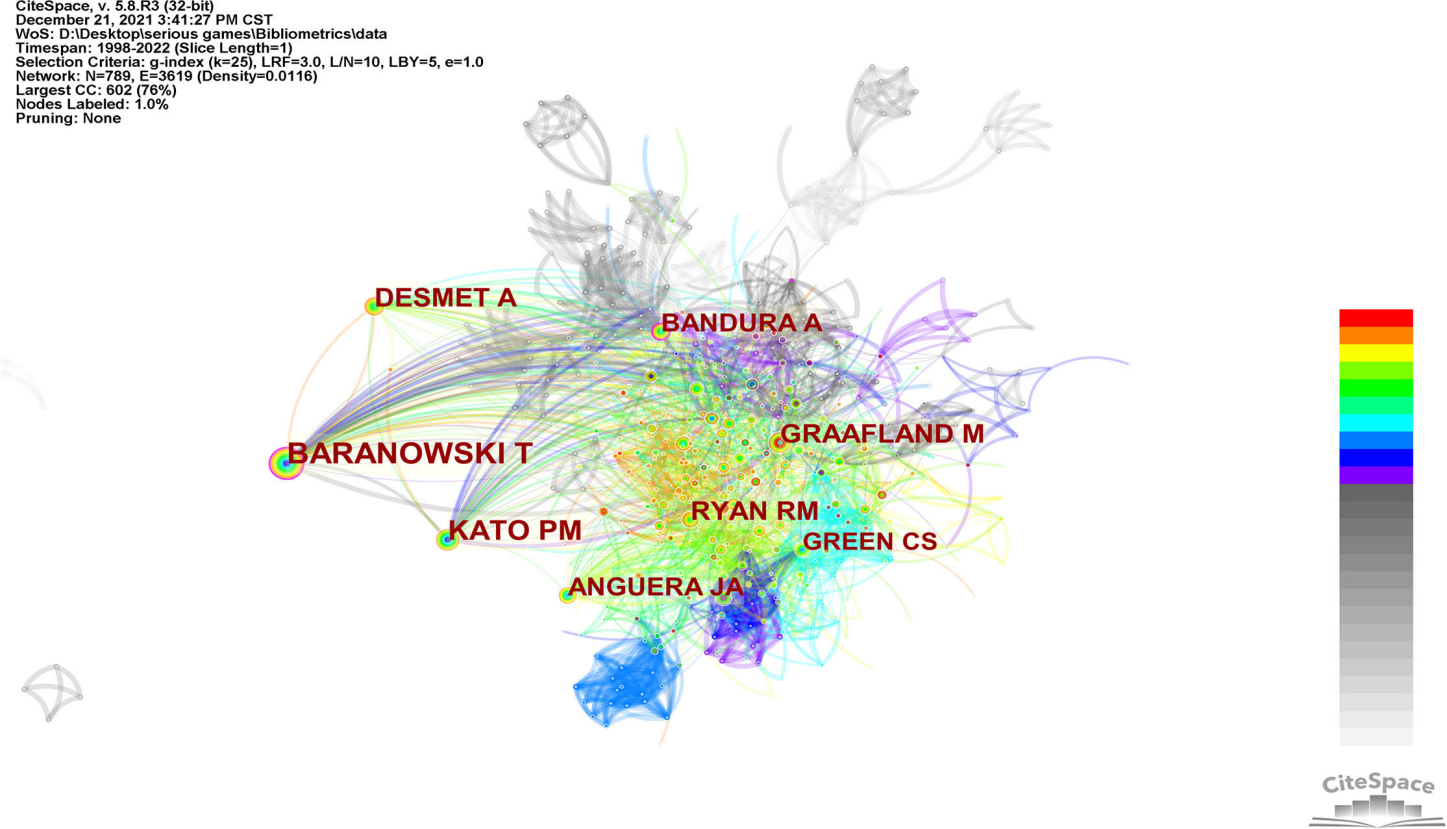
Author collaboration network.

As shown in Figure 3, different colors represent different years in which the author was cited. The larger the node, the more the author is cited, and the greater the influence. We can see that Baranowski T is the most influential author (Figure 3). It is obvious that there is purple outer ring outside the nodes of Baranowski T and Bandura A which means they have stronger correlation with other authors and more important influence. The lines between different nodes represent authors' cooperation, and the thicker the line is, the closer the cooperation is. The cited authors network indicates that the cooperation is close and academic atmosphere is strong in this field through the number of nodes (*N* = 789), the number of lines between nodes (*E* = 3,619), and the network density (Density = 0.0116).

The top 10 influential authors are listed in Table 2. Baranowski T has absolute priority with 103 citations, followed by Kato PM (*n* = 73 citations) and Desmet A *(n* = 58 citations). Although Bandura A is not cited many times (*n* = 50 citations), the centrality ranks second, indicating that he has great influence.

**Table 2:** The top 10 influential authors.

**Number**	**Cited Author**	**Year**	**Citations**	**Centrality**
1	Baranowski T	2009	103	0.16
2	Kato PM	2007	73	0.08
3	Desmet A	2016	58	0.05
4	Graafland M	2015	57	0.02
5	Ryan RM	2010	52	0.08
6	Bandura A	2000	50	0.13
7	Anguera JA	2014	45	0.04
8	Green CS	2012	41	0.07
9	Cohen J	2010	38	0.03
10	Primack BA	2015	36	0.02

#### Countries' Collaboration

**Figure 4:**
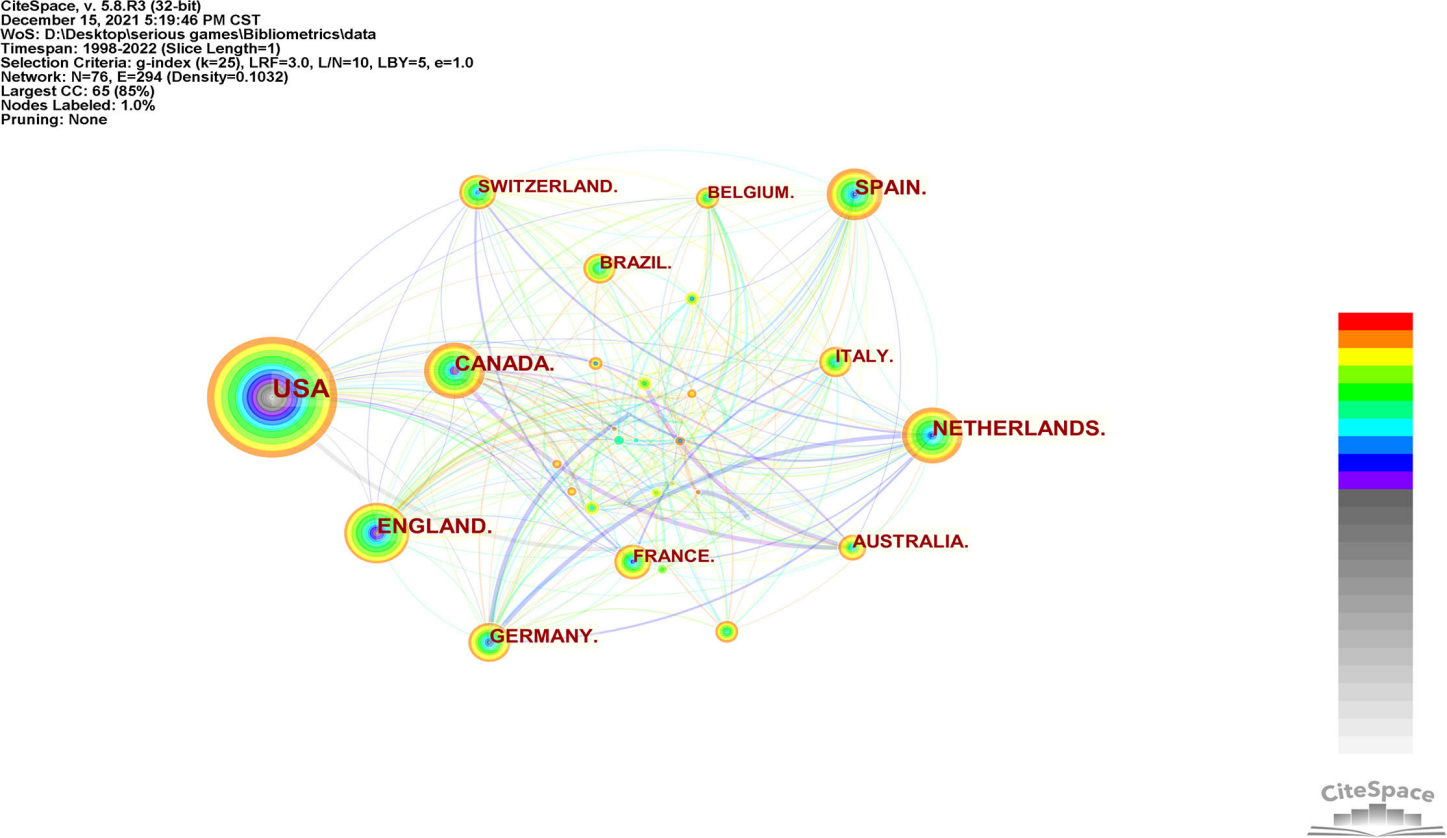
Countries' collaboration network.

Figure 4 shows a network visualization map of countries' collaboration. The node size is directly proportional to the number of articles issued by countries. It can be seen that the number of articles issued by the United States is absolutely dominant. The term “serious games” was first put forward by Abt who is an American (1), which means that the United States of America has become a country that studied and developed SGs earlier. Therefore, the number of documents issued by the USA is also in an absolute leading position (*n* = 214). The width of the line connecting different countries is directly proportional to the close cooperation between countries. The number of nodes is 76, the number of lines between nodes is 294, and the network density is 0.1032, showing that the cooperation between different countries is also very close, which is conducive to knowledge flow and discipline development.

#### Cited Journals

The top 10 cited journals publishing articles about SGs in health care were presented in Table 3. The top three journals with cited frequency were *Plos ONE* (*n* = 268 citations), *Games for Health Journal* (*n* = 209 citations), *Journal of Medical Internet Research* (*n* = 197 citations) with an impact factor in 2020 of 3.240, 3.204, 5.428, respectively. The impact factors of two journals are more than 50, namely, *JAMA* (IF = 56.274) and *Lancet* (IF = 79.323). The journal, *JAMA*, is not cited frequently, but it ranks first in the centrality, indicating that it has a high influence in serious studies in health care. Seven of the top 10 cited journals were published in the United States, which once again proves the influence of American articles in this field.

**Table 3:** The top 10 cited journals.

**Number**	**Journal**	**Count**	**Centrality**	IF*2020
1	*Plos One*	268	0.01	3.240
2	*Games for Health Journal*	209	0.01	3.204
3	*Journal of Medical Internet Research*	197	0.02	5.428
4	*Computers in Human Behavior*	158	0.07	6.829
5	*JMIR Serious Games*	155	0.01	4.143
6	*Pediatrics*	155	0.07	7.125
7	*American Journal of Preventive Medicine*	135	0.08	5.043
8	*JAMA-Journal of the American Medical Association*	134	0.08	56.274
9	*Lancet*	125	0.01	79.323
10	*Archives of Physical Medicine and Rehabilitation*	123	0.06	3.966

#### Cited Articles

The top 10 cited articles were presented in Table 4. The most cited article, “A meta-analysis of serious digital games for healthy lifestyle promotion,” was published in *Preventive Medicine* in 2014 and received the highest citation (*n* = 27 citations). Among the 10 articles, 9 were reviews, of which 7/9 were systematic reviews, mainly involving the application of SGs in the field of health care. The rest was a clinical trial, which used SGs as an intervention to train the cognitive control ability of the elderly. Except that the article, a systematic literature review of empirical evidence on computer games and SGs, was published in non-medical journal (*Computers and Education*), the rest were published in medical related journals. Most of the highly cited and high-quality articles are reviews, indicating that the quality of some clinical studies related with SGs is not very high and needs to be further explored.

**Table 4:** The top 10 cited articles.

**Number**	**Title**	**Year**	**Count**	**Burst**	**Centrality**	**Journal**
1	A meta-analysis of serious digital games for healthy lifestyle promotion	2014	27	8.61	0.06	*Preventive Medicine*
2	A systematic review of serious games in training health care professionals	2016	21	7.32	0.02	*Simulation in Healthcare*
3	video game training enhances cognitive control in older adults	2013	20	6.07	0.07	*Nature*
4	Serious games for improving knowledge and self-management in young people with chronic conditions: a systematic review and meta-analysis	2016	19	3.43	0.14	*Journal of the American Medical Informatics Association*
5	A systematic review of serious games in medical education: quality of evidence and pedagogical strategy	2018	18	6.38	0.01	*Medical Education Online*
6	Serious gaming and gamification education in H health professions: systematic review	2019	16	5.76	0.01	*Journal of Medical Internet Research*
7	Games for health for children—current status and needed research	2016	15	4.03	0.01	*Games for Health Journal*
8	The benefits of playing video games	2014	15	5.73	0.01	*American Psychologist*
9	A systematic literature review of empirical evidence on computer games and serious games(effect)	2012	15	6.45	0.00	*Computers and Education*
10	Serious games for mental health: are they accessible, feasible, and effective? A systematic review and meta-analysis	2017	14	4.93	0.01	Frontiers in Psychiatry

#### Keywords

The top 20 keywords of the co-occurrence frequency are presented in Table 5. Among the top 20 keywords, “serious game,” “video game,” “virtual reality,” “game,” “simulation,” and “health,” “care” are the search terms in this articles. They belong to the basic vocabulary in this research field and do not have research characteristics, so they are not included in the analysis of keywords. All the remaining keywords with research significance are classified into the following three categories: One is the target group, including “children,” “adolescent,” “older adult,” and “adult,” the other is the research type, including “intervention” and “meta-analysis,” and the other is the research theme, including “physical activity,” “performance,” “rehabilitation,” “education,” “exercise,” and “design.”

**Table 5:** The top 20 keywords of the co-occurrence frequency.

**Number**	**Keyword**	**Count**	**Centrality**
1	Video game	98	0.07
2	Health	88	0.08
3	Children	87	0.22
4	Virtual reality	69	0.07
5	Physical activity	69	0.14
6	Adolescent	65	0.10
7	Intervention	63	0.03
8	Performance	58	0.09
9	Rehabilitation	51	0.05
10	Serious game	51	0.00
11	Older adult	47	0.07
12	Education	45	0.08
13	Meta analysis	41	0.05
14	Exercise	40	0.10
15	Technology	37	0.03
16	Game	37	0.04
17	Adult	36	0.12
18	Care	35	0.05
19	Simulation	35	0.03
20	Design	34	0.06

**Figure 5:**
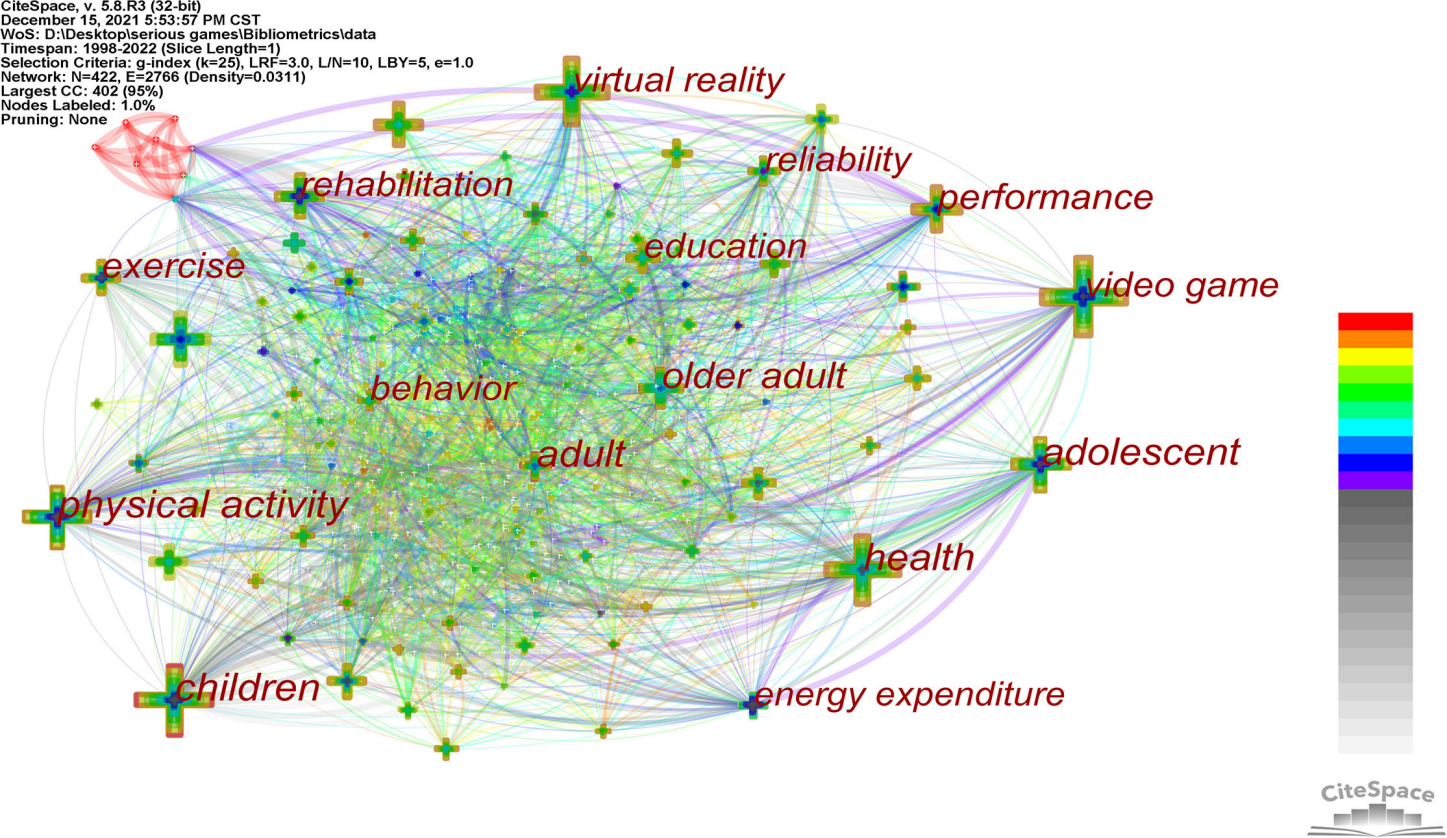
Keywords co-occurrence network.

Figure 5 shows a keywords co-occurrence network diagram. Different nodes represent different keywords, and node size is directly proportional to the co-occurrence frequency. The line between different nodes represents the co-occurrence relationship between different keywords. The thicker the line, the stronger the co-occurrence relationship. The number of nodes is 422, the number of lines between nodes is 2,766, and the network density is 0.0311, indicating that the research of SGs in health care is multi-theme.

## Dissuasion

### Knowledge Base

The co-citation refers to the relationship formed by two scientific articles cited by one or more later articles at the same time, which is a research method to measure the degree of relationship between articles, and first proposed by Small, an American information scientist, in 1973 (29). Therefore, articles with high cited frequency have a very important impact in a certain research field, forming knowledge base of this field (28). Therefore, we analyzed the academic basis of SGs in the field of health care through the top 10 cited articles.

Except for one clinical trial, other article types fall under the category of reviews, of which seven are systematic reviews. As an intervention, SGs have been carried out by researchers in various clinical trials. Therefore, a large number of systematic reviews or reviews have emerged. Most articles summarized the effectiveness of SGs on healthy lifestyle promotion (3), knowledge improvement (10), training for health care professionals (30), diagnosis, evaluation and treatment (11), and so on. Special games are a subcategory of digital games; however, the negative effects of traditional digital games have been reported, such as addiction, aggression (31), a series of psychological problems, and mental symptoms (e.g., anxiety and depression) (32, 33). There are main differences between SGs and traditional digital games: (1) The stakeholders: The stakeholders of SGs have increased people related with specific areas, such as healthcare providers, patients, and their families. (2) The primary goals: Special games are committed to accomplishing characterizing goal such as training and education, and traditional digital games just for pure entertainment. (3) Interest: Special games are mostly non-profit purposes, whereas most traditional digital games are for profit purposes. (4) Basic principles of design: Special games take the balance between serious parts such as education and entertainment factors; and entertainment parts such as the design principle, while traditional digital games do not consider serious parts.

### Research Hotpots and Trends

This study is an attempt of understanding research hotspots of SGs in health care and analyzing the development trends by extracting keywords. The top 20 keywords can be divided into the following four categories: Retrieval related words, research participants, research types, and research themes. Combined with the theme induction of scoping review and keyword co-occurrences of bibliometrics analysis, it is discussed from the following three aspects: Target groups, research themes, and game design.

#### Target Groups

Special games are widely used in different age groups, such as children, adolescents, youth, adults, and the elderly. According to the results of scoping review and bibliometric analysis, SGs are popular among children, adolescents, and the elderly. In children and adolescents, the application of SGs focuses on their mental health. Anxiety and depression are prominent mental health disorders among children and adolescents, with the high prevalence of 6.5% (anxiety) and 2.6% (depression) at a certain time (34) and females reporting rates higher than males (35). Compared with the traditional digital games that may lead to anxiety and depression, SGs have been proved to be reliable solutions (36, 37). Martinez et al. (38) conducted a systematic review of application for anxiety and depression in SGs for children and adolescents and then made suggestions that the application of SGs to anxiety and depression should be extended to all ages and the development and evaluation of SGs need to be standardized. Special games were also applied to increasing cancer knowledge, improving behavioral outcomes in adolescents and young adults with cancer (39), and improving type 1 diabetes self-management care in children and adolescents. A possible explanation that SGs can change children and adolescents' behaviors is that the characteristics of SGs (e.g., interesting, visual, and immersive) are enough to attract children and adolescents who also have strong learning and imitation ability (40).

In the elderly, SGs may have the potential to maintain capacity and increase self-efficacy in dementia, maintain posture balance, increase muscle strength (41), and improve treatment compliance (42). Due to the decline of physical function of the elderly, SGs mainly improve their physical activity indicators. Special games have multiple effects in different age groups. Therefore, targeting the specific ages, it is necessary to develop SGs with psychological or disease characteristics in future research.

#### Research Themes

According to the results of scoping review and bibliometric analysis, we have extracted the following two main themes: Rehabilitation and medical education. With the spread of coronavirus disease 2019 (COVID-19) pandemic, wearing masks, maintaining social distance, and quarantine are widely used interventions (43), which are the factors that prevents patients from face-to-face rehabilitation and medical students from face-to-face learning. Therefore, the need for innovative ways, such as telemedicine and distance education, to provide rehabilitation to patients and education to medical students has become an urgent call. Special games could be used as one of the means of telemedicine and distance education that can not only avoid the risk of contagion, but also monitor various indicators of rehabilitation and education.

#### Applying in Rehabilitation

Rehabilitation was defined as a process of restoring and rebuilding lost functions generally with physical activities or exercises which may be dull for patients (44, 45). So more interested methods, such as SGs, have emerged. Special games could improve treatment compliance in rehabilitation sessions and be divided into the following two categories: One was for cognitive rehabilitation, and the other was for physical rehabilitation (44). In cognitive rehabilitation, SGs can significantly improve fast visual processing and spatial working memory in patients with amnestic mild cognitive impairment (46). The research team of Rui et al. reviewed the application of SGs in cognitive rehabilitation (47), and then developed a SG web platform for cognitive rehabilitation (48). As a matter of fact, SGs are more used in physical rehabilitation compared with cognitive rehabilitation. For example, a randomized controlled trial showed that SGs can improve daily living physical functions in multiple sclerosis, such as movements speed, upper limb dexterity, and so on. The patients with stroke and cerebral palsy used SGs as a rehabilitation tool to improve clinical outcomes (49). However, most studies about SGs for rehabilitation lack the methodological quality (50), a standard framework needs to be developed imperatively.

#### Applying in Medical Education

With the development of science and technology, the means of medical education are constantly enriched, among which SG is a promising educational method. Special games were applied to operation, anesthesia, cardiopulmonary resuscitation, first aid, and so on. in medical education. Modern theories hold that learning is most effective in interesting, active, problem-based, and with feedback situations; and SGs are in line with this theory (51). There is an evidence that SGs can improve medical workers or students' knowledge (52), awareness of competition and cooperation (53), medical decision-making ability (54), and practical operation ability (55) in safe surroundings. Although many scholars apply SGs to medical education, the evidence of their effectiveness is only moderate (8). The problem still exists is how to balance the relationship between “entertainment” and “learning” in education (56), which is what game designers and educators need to think about in the future.

Consistent with the results of Trinidad M et al. (57), we all hold the opinion that physical rehabilitation and medical education are dynamic domains of SGs application, so there are related articles have increased exponentially.

#### Design of SGs

Many researchers have designed SGs for specific groups and they need to acquire design elements of SGs. Whyte et al. (58) examined the important design elements as listed in the following: Storyline, goals, feedback and rewards, difficulty and individuation, and choice. When designing SGs, the researchers also need a good basic framework. Johnsen et al. (59) designed SGs for nursing students based on the framework, namely task, users, representation, and function (TURF) and were developed by Zhang et al. (60). Graafland et al. (61) proposed a consensus-based framework which contained 62 items in 5 themes (namely, game description, rationale, functionality, validity, and data safety) for the assessment of specific medical SGs, and Epstein et al. (62) proposed a game design framework too. Although many frameworks for SGs have been proposed, the degree of application is not high and effectiveness needs to be verified. To improve the effectiveness of SGs, the design process is not just a matter for game developers alone, but requires multidisciplinary teamwork, considering all stakeholders [game designers, researchers, players and their families, policy makers, publishers and so on, (63)]. Therefore, the design elements of SGs, the implementation framework, and the characteristics of stakeholders should be taken into consideration in the design phase of SGs.

In the rapidly developing electronic era, SGs are increasingly playing a greater part in the field of health care, nevertheless, SGs also have some development resistance. At the macro level, the development cost of SGs is a major problem in low- and middle-income countries, and the market is more inclined to develop entertainment games and more commercial interests (64). Second, when designing SGs, the medical workers or the educators often ignore a very important factor in developing games—the fun, resulting in a situation where players have no interest and SGs ultimately are failing to work (65). For novices, SGs may lead to distracting and impeding learning (66). These existing problems could not be ignored and should be noticed by the policy makers and the game developers.

The results of our study yield some implications for designing and applying SGs in health care. For researchers: They can better understand what they need to focus on in the future, such as rehabilitation and medical education, and they can learn what frameworks can be used as a basis for evaluation when implementing SGs in health care through our results. For designer of SGs: They can refer to our results to think more about the balance between the serious parts and entertainment parts. For policy makers: They can better understand the importance and significance of the application of SGs in health care and think about what measures to take to promote the application of SGs in health care. For players: They can clearly understand the difference between SGs and traditional digital games, and make more rational use of SGs through our results.

## Conclusions

This study has presented an overview of the application status of SGs and put forward the research hotspots and trends. The continuity and development of COVID-19 has provided an opportunity for medical workers and educators to develop telemedicine and distance education of which SGs could be effective means, that could be paid more attention by researchers in the future. The SGs applications are growing up in health care, but still with some problems, consequently, a better and more applicable framework needs to be proposed and more and higher quality randomized controlled trial should be carried out.

### Strengths and Limitations

The strengths of our study include that we obtained eligible literature from WoS with high-quality articles and comprehensively analyze the status and trends of SGs in health care through the combination of qualitative analysis and quantitative analysis, focusing different bibliometric indicators. Simultaneously, there are some limitations as follows: First, only the WoS core database is retrieved in which the literature is skewed toward English journals and therefore documents in Chinese, Japanese, and other languages were underestimated. Second, we just summarized hundreds of articles without making a more in-depth detailed analysis, and future research can be further explored.

## Data Availability Statement

The original contributions presented in the study are included in the article/supplementary material, further inquiries can be directed to the corresponding author/s.

## Author Contributions

YW: writing—original draft. ZW and GL: writing—review. ZYW, QW, and WG: rechecking. YY, JW, and YZ: screening literature. XK and ZZ: checking the manuscript. LJ: revising language and content. XP: conceptualization and providing suggestions. All authors contributed to the article and approved the submitted version.

## Funding

The authors declare that this study received funding from Key Research Topics of Education and Teaching Reform in Tianjin University of Traditional Chinese Medicine with Grant No. 2016JYC04, Education and Scientific Research Project in 2021 of the 14th Five Year Plan for Higher Education of Traditional Chinese Medicine with Grant No. DZ-20-07, Tianjin Lifelong Education Research Program with Grant No. 202116 and Scientific Research Project of Tianjin Education Commission with Grant No. A201006303.

## Conflict of Interest

The authors declare that the research was conducted in the absence of any commercial or financial relationships that could be construed as a potential conflict of interest.

## Publisher's Note

All claims expressed in this article are solely those of the authors and do not necessarily represent those of their affiliated organizations, or those of the publisher, the editors and the reviewers. Any product that may be evaluated in this article, or claim that may be made by its manufacturer, is not guaranteed or endorsed by the publisher.
